# Competencies influencing childcare providers' infectious-disease prevention in routine and outbreak contexts: a study in South Korea

**DOI:** 10.3389/fpubh.2025.1707317

**Published:** 2026-01-14

**Authors:** Won-Oak Oh, Yoo-Jin Heo, Myung Jin Jung, Jihee Han, Eunji Lee

**Affiliations:** 1 College of Nursing, Korea University, Seoul, Republic of Korea; 2 College of Nursing, Dankook University, Cheonan, Republic of Korea; 3 Department of Nursing, Jungwon University, Goesan-gun, Chungcheongbuk-do, Republic of Korea; 4 University of Maryland School of Nursing, Baltimore, MD, United States

**Keywords:** childcare providers, community health, early childcare settings, health promotion, infection-prevention competence, infectious-disease prevention practice, outbreak response

## Abstract

**Introduction:**

Empirical evidence is limited regarding the competency domains that influence childcare providers' routine and outbreak-related infection-prevention behaviors in early-childhood settings. This study, therefore, aimed to identify the competencies that affect the infectious-disease prevention behaviors of childcare providers.

**Methods:**

A cross-sectional descriptive study was conducted with 239 childcare providers in an early-childhood education center. The variables were measured using self-administered questionnaires. Descriptive data for each factor were assessed, and Pearson's correlation analysis was used to analyze the correlations between infectious-disease prevention practices and the related factors. Stepwise multiple regression was used to identify the predictive factors for infectious-disease prevention practices. Data were collected between May 31, 2023, and June 12, 2023.

**Results:**

The factors influencing childcare providers' daily infectious-disease prevention practices were educational guidance (β = 0.19, *p* = 0.02), environmental management (β = 0.22, *p* = 0.001), and surveillance (β = 0.17, *p* = 0.022) competencies; these accounted for 53.7% of the variance. The factors influencing infectious-disease outbreak prevention practices were surveillance (β = 0.22, *p* = 0.011), research and empowerment (β = 0.20, *p* = 0.032), and personal management (β = 0.18, *p* = 0.031) competencies; these accounted for 43.9% of the variance.

**Discussion:**

Enhancing childcare providers' infectious-disease prevention practices requires providing them with educational information, internal motivation, and support for effective strategies tailored according to children's ages. This study's findings can help community health managers and policymakers develop interventions and policies to support the prevention and management of infectious diseases in young children.

## 1 Introduction

Owing to changes in social structures and increases in parents' economic activities, many preschool children now receive care in childcare centers or kindergartens (1). In Organization for Economic Co-operation and Development (OECD) countries, on average, 29% of children aged 0–2 and 85% of those aged 3–5 are enrolled in early-childhood education programs (2). In the United States, 57.5% of children aged 3–5 years receive center-based care (1). In Korea, meanwhile, 85.6% of the preschool-age population attended kindergartens or daycare centers in 2025 (3)—a rate that has been steadily increasing (2, 3).

Infancy and preschool years represent a period of rapid physical growth and foundational development (4), during which effective health management is critical for lifelong wellbeing (5–7). Young children are especially vulnerable to illness because of their immature immune systems and limited ability to recognize or communicate health concerns (4). Furthermore, childcare settings—where a large proportion of young children spend considerable time (2)—facilitate the rapid transmission of infectious diseases, such as the common cold, hand-foot-and-mouth disease (HFMD), and emerging pathogens, owing to the high-density group environments (8–10). Recent outbreaks further highlight these risks: For instance, a 2023 norovirus outbreak in a Korean childcare facility demonstrated the prominence of person-to-person transmission (11), while concerns about emerging pathogens such as Kingella kingae continue to grow (12). Outbreaks such as the large-scale HFMD epidemic in France (13) also underscore the international relevance of this issue. In light of such phenomena, infection control in childcare settings is essential, underscoring the critical role played by childcare providers (8).

Since the COVID-19 pandemic, infection prevention and control programs have become essential components of early-childhood education and care (14). The core competencies expected of childcare providers now encompass areas such as hand-hygiene practices, symptom monitoring, the continuous updating of infection-related information, improved decision-making, and strengthened collaboration with health authorities (15, 16). As infectious-disease risks persist and post-pandemic conditions evolve, the professional competencies of childcare providers are more important than ever for ensuring safe and healthy learning environments (9, 17–19).

Despite childcare providers' pivotal role in safeguarding children's health, much of the existing literature focuses on infection prevention in clinical settings or schools that serve older children (20). Research on childcare providers has examined infection-related knowledge, perceived educational needs, or simple correlations among these variables (19, 21). Interventions often focus on improving infection-control knowledge or hand-hygiene practices (15). Since the COVID-19 pandemic, childcare providers have faced higher expectations, including the ability to collaborate with medical experts, make rapid decisions, and incorporate up-to-date infectious-disease information (16). Nevertheless, research tends to narrowly focus on behaviors such as handwashing or knowledge acquisition (19, 21).

The Association for Professionals in Infection Control and Epidemiology (APIC) established a competency model that provides a standardized framework detailing the knowledge, skills, and performance domains necessary for infection prevention (22). The APIC model conceptualizes infection-prevention behavior as a multidimensional construct encompassing domains such as leadership and program management, infection prevention and control, technical skills, and performance improvement/implementation science (22). Few studies have holistically examined these multidimensional competencies (15, 23–28). Moreover, everyday preventive practices in childcare settings differ from outbreak-response practices, which require more urgent and intensive action (15, 16). Despite this distinction, few studies have separately analyzed the factors influencing infection-prevention behaviors in routine vs. outbreak contexts. Such differentiation is essential for developing tailored educational programs and policies that address the unique demands of each context. This study, therefore, aimed to differentiate between routine and outbreak-related infection-prevention behaviors among childcare providers and identify the distinct factors associated with each. This approach can provide meaningful evidence to help strengthen infection-prevention practices in early-childhood settings and inform targeted policy interventions.

### 1.1 Problem statement

Childcare facilities are spaces where children in the same age group are densely concentrated, creating high-risk environments for the rapid spread of respiratory and waterborne infectious diseases (29, 30). Over 36% of all waterborne and foodborne infectious-disease outbreaks occur in childcare facilities, representing the second-highest incidence rate (31). Additionally, major respiratory infections such as tuberculosis and pertussis have shown sharp upward trends in childcare centers and kindergartens over the past 5 years (31–33). Although various education programs have been implemented, significant gaps persist between infection-prevention education and actual practice (15, 21). In Korea, only childcare facilities with 100 or more children are required to have professional medical personnel (34); this leaves infection-prevention management largely dependent on childcare providers in most facilities. Therefore, to improve childcare providers' infection-prevention practices, it is necessary to investigate the diverse situational factors influencing prevention behaviors and identify existing competency gaps. Accordingly, this study analyzed how core infection-prevention competencies among childcare providers—identified using the APIC competency model—affect prevention behaviors in routine vs. outbreak situations.

### 1.2 Literature review

Childcare settings are high-risk environments for respiratory and enteric infections owing to close contact among young children. Group care has been shown to carry a twofold to threefold higher infection rate than home-based care (10), and respiratory and gastrointestinal infections remain common in these settings (35). Recent outbreaks such as large-scale HFMD in France (13) and renewed enterovirus circulation in Europe (36) further highlight post-pandemic vulnerability in early-childhood facilities.

Childcare providers play a central role in preventing infections in these environments (17). Their environmental management and daily preventive practices are critical determinants of transmission (27), and evidence shows that appropriate cleaning and disinfection procedures can significantly reduce microbial loads (27). Prior studies indicate that institutional support, professional training, communication skills, and self-efficacy contribute positively to infection-prevention behaviors (23, 24), while nursing knowledge remains a key predictor of infection-control performance (37). Educational interventions—such as hand-hygiene programs (26, 28), health literacy training (25), and comprehensive infection-control curricula (15, 38)—have shown improvements in knowledge and confidence.

Nevertheless, compliance with infection-control guidelines in childcare settings remains insufficient. A nationwide Japanese survey reported adherence rates below 70%, largely attributed to providers' limited experience and knowledge (16). Korean studies also highlight deficits in surveillance competency, particularly among less experienced providers (39). Since COVID-19, childcare providers have been expected to assume expanded roles, including collaboration with healthcare professionals, evidence-based decision-making, and the acquisition of emerging infectious-disease information (16). However, research continues to focus on individual behaviors such as knowledge acquisition or hand hygiene (19, 21).

The APIC competency model offers a multidimensional framework that can help us advance beyond these fragmented approaches (22). Building on the APIC model, Oh (40) identified eight infection-prevention competency domains for childcare providers, including environmental management, surveillance, educational guidance, and research and empowerment. Applying this framework enables a clearer identification of the specific competencies required for both routine prevention and outbreak response.

In summary, previous studies have largely examined isolated aspects of infection prevention, without simultaneously exploring how multiple competency domains influence childcare providers' routine and outbreak-related practices. We addressed this gap by using the APIC competency model to multidimensionally analyze childcare providers' infection-prevention competencies and identify the domains most strongly associated with actual preventive behaviors in early-childhood settings.

## 2 Materials and methods

### 2.1 Purpose

This study aimed to identify competency-focused factors that influence infectious-disease prevention practices among childcare providers.

### 2.2 Design and sample

Our descriptive survey aimed to identify competency-focused factors influencing infectious-disease prevention practices among childcare providers. The participants were childcare providers, including daycare and kindergarten teachers, with at least 6 months of work experience who were currently employed at early-childhood education institutions. We excluded individuals who were not directly involved in educational duties at early-childhood education institutions (e.g., administrative or management staff) or those exclusively caring for children with disabilities. A total of 239 participants were included, and data from all 239 participants were used in the final analysis.

### 2.3 Data collection and ethical considerations

To efficiently recruit participants and ensure the accuracy and reliability of the survey data, we conducted data collection in collaboration with a professional research company (Korea Research Corporation). This study was conducted as an online survey using a panel managed by the research company. Data were collected between May 31 and June 12, 2023. At the start of the online survey, participants received a document that outlined the purpose of the research, assured confidentiality, emphasized that participation was voluntary, clarified the option to withdraw, and explained how their personal information would be managed. Participants were informed that they could withdraw from the study at any time.

### 2.4 Measures

#### 2.4.1 Demographic characteristics

A 10-item questionnaire was used to collect data on the general characteristics of childcare providers working in early-childhood education centers. The questionnaire included items related to gender, age, marital status, educational background, work experience, type of early-childhood education institution, type of daycare center, size of daycare center, and experience with infectious-disease prevention education.

#### 2.4.2 Infectious-disease prevention self-efficacy

We used the infectious-disease prevention self-efficacy scale developed by Yang and Kwon (41) to measure self-efficacy in infectious-disease prevention among the childcare providers. This tool consists of 15 items rated on four-point Likert scales. Each item was scored from 1 (“not confident at all”) to 4 (“very confident”), with higher scores indicating greater self-efficacy for infectious-disease prevention. The Cronbach's alpha was 0.93.

#### 2.4.3 Educational needs for infectious-disease prevention

Educational needs for infectious-disease prevention among childcare providers were assessed using a questionnaire developed by the authors. An initial item pool was generated through a review of the literature on infectious-disease prevention in early-childhood settings and through expert consultation with an infection-control nurse, a pediatric infectious-disease specialist, and a childcare facility nurse with substantial experience in childcare health management. Based on these inputs, 14 preliminary items were drafted to address key educational domains, including major childhood infectious diseases, prevention and management strategies, and environmental and hygiene management.

To enhance content validity, all items underwent expert review, during which language clarity and item completeness were evaluated and refined. Prior to the main survey, two childcare teachers and two childcare center directors reviewed the items to assess readability, comprehensibility, and appropriateness for childcare settings. The items were scored on a four-point scale ranging from 1 (“not necessary at all”) to 4 (“very necessary”). The total scores were calculated, such that higher scores indicated a greater overall need for education on infectious-disease prevention.

#### 2.4.4 Infectious-disease prevention competency

To assess the infectious-disease prevention competency of the childcare providers, this study used the Infectious-Disease Prevention Competency Scale for Childcare Providers, which was developed by the researchers (40). This self-report scale comprises 38 items divided into eight domains: leadership and proficiency, environmental management, knowledge and documentation, educational guidance, surveillance, infection risk management, research and continuing education, and personnel management. The scale uses five-point Likert scales, with each item rated from five points for “strongly agree” to one point for “strongly disagree.” Higher scores reflect greater competency in preventing infectious diseases. The Cronbach's alpha was 0.93.

#### 2.4.5 Infectious-disease prevention practice

Infectious-disease prevention practice was assessed using a tool developed by the researchers. The tool is based on 32 strategies proposed by Coronado et al. (42) for use in childcare and educational environments to prevent SARS-CoV-2 transmission. These strategies reflect CDC recommendations for preventing infectious diseases and include content on everyday actions, actions when someone is ill, and communication and support related to preventive practices. Because the original checklist was in English, we followed a standardized forward–backward translation procedure. Two nursing professors who were fluent in English independently performed forward translations of the original items into Korean, and the two versions were reconciled into a single Korean version through discussion among the researchers. Back-translation was then performed and compared with the original to verify semantic equivalence, and discrepancies were resolved through iterative revision. During the adaptation process, four items specific to COVID-19 practices (e.g., mandatory mask wearing and social distancing), which held less relevance for ongoing general infectious-disease prevention in childcare settings, were removed. Based on a literature review, we added four items related to the prevention of contact-transmitted infections to modify and supplement the tool while maintaining a total of 32 items. The preliminary Korean version was then reviewed by a childcare facility nurse, an infection-control nurse, a pediatric infectious-disease specialist, and a nursing professor to evaluate language clarity, content relevance, and completeness. Subsequently, two childcare teachers and two childcare center directors reviewed the items to assess readability, comprehensibility, and appropriateness for childcare settings, leading to minor refinements. Responses were scored on a four-point scale from 1 (“never do it”) to 4 (“always do it”). Total scores were calculated across the 32 items, with higher scores indicating a higher level of infectious-disease prevention practice.

### 2.5 Sample size determination

Sample size was determined using G^*^Power 3.1.9.2. The parameters were set for multiple regression analysis, with a significance level of 0.05, a power of 0.95, an effect size of 0.15, and 14 predictors (10 general characteristics and four independent variables). An effect size of 0.15 was derived from a previous study (37) of childcare providers' infectious-disease prevention practices during the pandemic. This calculation resulted in a minimum sample-size requirement of 194 participants (43). To account for a potential dropout rate of 20%, the target sample size was set at 239 participants.

### 2.6 Data analysis

Data were analyzed using SPSS/WIN 26.0. Descriptive statistics, such as frequency, percentage, mean, and standard deviation, were used to analyze the general characteristics of the participants. We analyzed the extent of infectious-disease prevention practices and the factors influencing them. The analysis included the minimum, maximum, mean, and standard deviation. Pearson's correlation analysis was used to analyze the correlation between infectious-disease prevention practices and the related factors. The level of infectious-disease prevention practices was analyzed based on general characteristics using *t*-tests and one-way analysis of variance. Stepwise multiple regression identified the predictive factors for the prevention of infectious diseases. Our regression analyses included variables that, based on our literature review, were expected to influence infection-prevention practices—namely, childcare providers' self-efficacy, educational needs, and infection-prevention competencies derived from the APIC model—along with general childcare provider characteristics that showed significant correlations in the preliminary analysis. To assess multicollinearity, we checked tolerance and variance inflation factors (VIFs). A variable is considered to have multicollinearity if tolerance is less than 0.1 or the VIF value is greater than 10. The internal consistency and reliability of the tools were evaluated based on Cronbach's alpha.

### 2.7 Ethics considerations

This study was approved by Korea University's Institutional Review Board (IRB no. KUIRB-2023-0119-01). All procedures were carried out in accordance with the Declaration of Helsinki. Participants received the study purpose statement, information sheet, consent form, and questionnaire pack online. The information sheet and consent form were signed before data collection.

## 3 Results

### 3.1 Demographic characteristics and differences in infectious-disease prevention practices according to demographics

Data from all 239 participants were used for the final analysis, owing to simultaneous responses. Table 1 presents the participants' general characteristics. Childcare providers who received infectious-disease prevention education indicated a greater extent of infectious-disease prevention practices than those who did not receive such education (*p* = 0.016).

**Table 1:** Demographic characteristics of study participants (*N* = 239).

**Characteristics**	**Categories**	***N* (%)**	***t* or *F***	***p-*value**
Age	20–29 years	21 (8.8)	0.44	0.724
	30–39 years	60 (25.1)		
	40–49 years	95 (39.7)		
	Over 50 years	63 (26.4)		
Marital status	Single	55 (23.0)	−0.77	0.441
	Married	184 (77.0)		
Parenting experience	None	75 (31.4)	−0.1	0.918
	Yes	164 (68.6)		
Qualifications	Childcare provider	155 (64.9)	−0.51	0.614
	Kindergarten teacher	84 (35.1)		
Education	Completion of childcare provider training course	10 (4.2)	1.39	0.248
	College graduate	84 (35.1)		
	Bachelor's degree	119 (49.8)		
	Graduate school or higher	26 (10.9)		
Work experience	Less than 5 years	45 (18.8)	1.51	0.2
	5–9 years	72 (30.1)		
	10–14 years	74 (31.0)		
	15–19 years	26 (10.9)		
	20 or more years	21 (8.8)		
Type of childcare setting	Public	99 (41.4)	0.58	0.627
	Private	98 (41.0)		
	Home-based	34 (14.2)		
	Other	8 (3.3)		
Size of childcare setting	Less than 10	9 (3.8)	0.75	0.557
	10–29 people	83 (34.7)		
	30–49 people	50 (20.9)		
	50–99 people	61 (25.5)		
	100 or more	36 (15.1)		
Previous education in infectious disease prevention	Yes	175 (73.2)	2.45	0.016^*^
	No	64 (26.8)		

### 3.2 Study variables of childcare providers

Table 2 presents the descriptive statistics of the variables related to infectious-disease prevention practices and the Cronbach's alpha for each questionnaire. The average score for childcare providers' infectious-disease prevention practices was 112.51, while the average score for infectious-disease prevention competency was 159.42. Average self-efficacy in infectious-disease prevention was 51.94, and the average need for education on infectious-disease prevention and management was 51.33.

**Table 2:** Descriptive statistics for the study variables (*N* = 239).

**Variables**	**Mean (SD)**	**Rage (Min–Max)**	**Cronbach's alpha**
Self-efficacy in preventing infectious diseases	51.94 (6.12)	23.0–60.0	0.93
Demand for education on infection prevention and management	51.33 (5.47)	37.0–56.0	0.91
Competency in preventing infectious diseases	159.42 (20.26)	107.0–190.0	0.92
Leadership and proficiency	15.55 (2.57)	8.0–20.0	
Environmental management	22.90 (2.20)	15.0–25.0	
Knowledge and documentation	16.82 (2.51)	9.0–20.0	
Surveillance	20.21 (3.67)	11.0–25.0	
Infection risk management	17.68 (2.29)	11.0–20.0	
Educational guidance	26.21 (3.17)	18.0–30.0	
Research and empowerment	23.77 (4.35)	12.0–30.0	
Personal management	16.30 (2.83)	6.0–20.0	
Infectious disease prevention practices	112.51 (14.45)	71.0–128.0	0.90
Everyday prevention practice	48.05 (4.59)	33.0–52.0	
Disease outbreak practice	41.27 (6.67)	17.0–48.0	
Communication and support practice	23.18 (5.09)	7.0–28.0	

### 3.3 Correlations among study variables

The scores for childcare providers' practices in infectious-disease prevention, everyday prevention practices, and prevention practices during disease outbreaks were significantly correlated with self-efficacy in infectious-disease prevention (*p* < 0.001), the need for education on infectious-disease prevention and management (*p* < 0.001), the total score for infectious-disease prevention competency (*p* < 0.001), and the scores for subcompetencies in infectious-disease prevention (*p* < 0.001; Supplementary Table S1).

### 3.4 Factors affecting childcare providers' infectious-disease prevention practices

Stepwise regression was conducted to identify the factors influencing infectious-disease prevention practices among childcare providers. We analyzed the correlations among the independent variables when reviewing the basic assumptions for the regression analysis. We included statistically significant general characteristics, such as whether individuals had received infectious-disease education, and variables that showed a significant relationship with infectious-disease prevention behaviors in the model. The Durbin–Watson statistic indicated no autocorrelation in the regression for infectious-disease prevention practice at 1.957, everyday prevention practice at 1.982, prevention practices during disease outbreaks at 2.075, and communication and support practices at 1.768. Tolerance was between 0.285 and 0.762, which is above the threshold of 0.1. VIF ranged from 1.313 to 3.672, which is below the critical value of 10. Therefore, multicollinearity was not an issue (Table 3).

**Table 3:** Factors affecting infectious disease prevention practice (*N* = 239).

**Variables**	***B***	**S.E**.	**β**	***t***	**95% CI**	***p*-Value**	**Tolerance**	**VIF**
Competency-educational guidance	1.45	0.33	0.32	4.38	(0.80, 2.10)	<0.001	0.38	2.64
Demand for education on infection prevention and management	0.66	0.14	0.25	4.59	(0.37, 0.94)	<0.001	0.68	1.46
Competency-research and empowerment	0.95	0.22	0.29	4.29	(0.51, 1.39)	<0.001	0.45	2.24
*F* = 88.639, *p* < 0.001, Adjusted *R*^2^ = 0.525, *R*^2^ = 0.531								
**(a) Everyday prevention practice**								
Competency-educational guidance	0.28	0.12	0.19	2.35	(0.05,0.52)	0.020	0.29	3.51
Demand for education on infection prevention and management	0.26	0.05	0.31	5.47	(0.17,0.35)	0.000	0.61	1.65
Competency-environmental management	0.45	0.13	0.22	3.42	(0.19, 0.71)	0.001	0.49	2.04
Competency-surveillance	0.21	0.09	0.17	2.31	(0.03,0.38)	0.022	0.38	2.64
*F* = 70.055, *p* < 0.001, Adjusted *R*^2^ = 0.537, *R*^2^=0.545								
**(b) Disease outbreak practice**								
Competency-research and empowerment	0.31	0.14	0.20	2.16	(0.03,0.59)	0.032	0.27	3.67
Demand for education on infection prevention and management	0.24	0.07	0.20	3.56	(0.11,0.38)	0.000	0.76	1.31
Competency-surveillance	0.39	0.15	0.22	2.55	(0.09,0.69)	0.011	0.33	3.03
Competency-personal management	0.43	0.20	0.18	2.17	(0.04, 0.82)	0.031	0.33	2.99
*F* = 47.646, *p* < 0.001, Adjusted *R*^2^ = 0.439, *R*^2^ = 0.449								
**(c) Communication and support practice**								
Competency-educational guidance	0.43	0.14	0.27	3.17	(0.16,0.70)	0.002	0.38	2.65
Competency-research and empowerment	0.32	0.09	0.28	3.50	(0.14, 0.50)	0.001	0.44	2.25
Demand for education on infection prevention and management	0.13	0.06	0.14	2.26	(0.02,0.25)	0.025	0.67	1.49
*F* = 32.259, *p* < 0.001, Adjusted *R*^2^ = 0.344, *R*^2^ = 0.355								

VIF, variance inflation factor.

Competencies in educational guidance and research and empowerment, as well as the need for education in infection prevention and management, were found to be factors affecting total infectious-disease prevention practices among childcare providers. Overall explanatory power was 52.5%, with the most influential variable being competency in educational guidance (β = 0.32, *p* < 0.001). Higher educational guidance competency was associated with the more consistent use of overall preventive behaviors among childcare providers. Everyday prevention practices are influenced by competencies in educational guidance, environmental management, and surveillance. Additionally, the need for education in infection prevention and management was the most influential variable (β = 0.31, *p* < 0.001), with an overall explanatory power of 53.7%. Higher demand for education on infection prevention and management and environmental management competency were associated with the more consistent use of everyday preventive behaviors among childcare providers. Competencies in research and empowerment, surveillance, personal management, and education in infection prevention and management are important factors in preventive practices during disease outbreaks. Competency in surveillance was the most influential variable (β = 0.22, *p* = 0.011), with an overall explanatory power of 43.9%. Higher competency in surveillance was associated with the more consistent use of preventive behaviors during outbreaks among childcare providers. Competencies in educational guidance and research and empowerment, as well as the need for education in infection prevention and management, were found to affect communication and support behaviors. The overall explanatory power of these factors was 34.4%, with competency in research and empowerment being the most influential variable (β = 0.28, *p* = 0.001; Table 3). Higher research and empowerment competency was associated with more consistent use of communication and support behaviors among childcare providers.

## 4 Discussion

### 4.1 Significance of the study

Early-childhood education and care settings are high-risk environments for infectious-disease transmission because young children live and interact in close proximity (30, 35). Recent data indicate a steady increase in waterborne, foodborne, respiratory, and contact-transmitted infections in childcare and similar facilities (36), and infection prevention has become a major policy priority in the post-COVID-19 era (44, 45). In this context, identifying the infection-prevention competencies of childcare providers addresses an urgent public health need to protect the health of children, families, and communities (16).

Our findings provide evidence-based insights that can help childcare providers, facility administrators, local public health centers, and health and education policymakers strengthen infection-prevention capacity in childcare settings. The findings can also inform the development of prevention guidelines, educational programs, and management strategies that can be applied in practice, ultimately helping reduce the incidence of infections in childcare centers and the related burden on families and communities.

This study additionally contributes academically by using the APIC competency model to measure multidimensional infection-prevention competencies and by distinguishing between providers' routine preventive behaviors and outbreak-response actions. Furthermore, our results provide clear direction for developing effective infection-prevention strategies in childcare settings. Training programs can prioritize strengthening key competencies such as educational guidance, surveillance, and research and empowerment to enhance practical prevention skills. The findings may also inform policy decision-making, including revisions to professional renewal requirements or in-service training programs to incorporate competency-based infection-prevention assessments.

This study examined the factors that influence infectious-disease prevention practices among childcare providers. The goal was to provide data for the development of interventions to improve infectious-disease prevention practices. We found that childcare providers' infectious-disease prevention practices were influenced by their need for education on infection prevention and management and their infectious-disease prevention competency—specifically, in educational guidance and research and empowerment. These factors accounted for 52.5% of the variance in infectious-disease prevention practices among childcare providers.

Competency in educational guidance had the most significant effect on practice. This finding aligns with previous research showing that providers with higher levels of infection-prevention understanding and practical teaching experience—particularly those with professional training—demonstrate better adherence to hand hygiene, environmental disinfection, and other preventive behaviors (21, 23, 24, 37). Educational guidance competency also significantly predicted everyday preventive practices. Notably, while this competency did not exert a significant effect on outbreak-response behaviors, it played a key role in routine prevention. This could be because the formation of consistent daily habits contributes more effectively to long-term infection control than short-term responses during acute events (46). During outbreaks, structural protocols such as isolation procedures and PPE use typically dominate the response, whereas in everyday settings, developmentally appropriate instruction by providers helps children internalize routines such as handwashing and respiratory etiquette, thereby preventing disease occurrence at its source (47, 48). This competency encompasses childcare providers' ability to deliver infection-prevention education using methods tailored to children's developmental stages (4). It also involves guiding the formation of preventive behavior habits in daily life, such as handwashing, toothbrushing, and coughing etiquette (49). Additionally, it involves providing appropriate information on infectious-disease management and prevention, not only for children but also for their families (17). To effectively prevent and manage infectious diseases, it is crucial to integrate disease prevention behaviors into children's daily lives as a natural and expected part of their routine (4). Roleplaying, simulation games, and repeated demonstrations of correct preventive behaviors—as well as educational methods that allow for direct observation and experience—can help establish these behaviors as habits (4). Additionally, enhancing cooperation with families for infection prevention and management is crucial. Indeed, previous studies have reported that the joint management of a child's safety and health by the family is more effective than unilateral efforts by childcare providers (40, 42). Thus, it is important to share information on infectious diseases and prevention strategies with children and their families.

Research and empowerment competency was the next most significant factor influencing infectious-disease prevention practices. This competency was found to significantly influence both outbreak-related preventive practices and communication and support behaviors. However, it did not show a significant effect on routine prevention. This could be because sustained learning efforts—such as maintaining interest in emerging infectious diseases, continuously updating knowledge, applying research findings to practice, and educating parents or colleagues—translate into research-informed decision-making and effective communication during crisis situations (50). In the context of an outbreak, when rapid judgment and action are required amid uncertainty, these competencies become critical determinants of a provider's response capacity (51). Therefore, research and empowerment competency is a key factor whereby ongoing learning investment in routine periods strengthens outbreak preparedness. Thus, the continued development of training programs that enhance these competencies is essential. This competency is closely linked to childcare providers' voluntary engagement in infection-prevention activities and their attitudes toward managing infectious diseases. Empowerment reflects the capacity to take proactive action driven by internal motivation, and such internalized motivation positively influences preventive practices, consistent with Joo and Kim's (37) findings. In this study, research and empowerment scores were lower than those in other competency domains, suggesting that current infection-control education for childcare providers—which mainly focuses on basic guidelines and information provision—might not adequately strengthen internal motivation. This highlights the need for training approaches that not only provide structural support (e.g., institutional policies, available resources, organizational support) but also cultivate childcare providers' intrinsic motivation and sustained engagement with emerging infectious-disease information.

Meanwhile, we found that competency in environmental management significantly affected infectious-disease prevention behaviors in daily life. This is consistent with a previous systematic review showing that everyday infection-prevention behaviors—such as toy sanitization, ventilation, and surface disinfection—are significantly influenced by environmental management competency (15). This suggests that effectively managing the physical environments of early-childhood education and care settings, where children live and interact, is essential for preventing the spread of infectious diseases. It is important to recognize environmental responsibility and childcare providers' role in maintaining a safe and healthy environment, free from infectious diseases. In contrast to previous studies (52), which reported a low level of knowledge and high demand for education in environmental management, environmental management had the highest average score (4.58) among the subareas of infectious-disease prevention competency. This could be related to childcare providers' increased awareness of environmental management following the pandemic. It is crucial to develop management strategies that consider children's age, as the environmental management aspects of childcare settings can vary depending on age. For instance, in facilities that cater to infants and toddlers, it is necessary to manage equipment, such as diaper changing areas, daytime nap pads, cribs, and related items (53). Therefore, infectious-disease prevention education for childcare providers should be differentiated based on the age and developmental level of the children in the facility.

In contrast to routine prevention—where environmental management emerged as a key factor—personal management competency played a significant role in the context of infectious-disease outbreaks. This could be because, in outbreak situations, staff-wide risk awareness, infection-control training, information sharing, and restrictions on external visitors are more important for preventing transmission than routine surface disinfection alone (50). This finding emphasizes that collaboration among staff members is central to outbreak containment in childcare settings.

Accordingly, infection-prevention training and organizational guidelines in childcare facilities should be differentiated based on the demands of routine vs. outbreak contexts. Routine prevention requires strengthening environmental management competencies, whereas outbreak situations call for enhanced personal management skills, rapid decision-making, and effective interstaff communication and collaboration. Developing competency-based training that reflects these contextual needs will improve infection-control efficiency and enable more timely, coordinated responses during outbreaks.

Our findings offer insights into the factors influencing childcare providers' infection-prevention practices and suggest directions for strengthening preventive behaviors in early-childhood settings. Based on the findings, community health managers and policymakers should design educational programs that enhance providers' intrinsic motivation and support the continuous acquisition and application of up-to-date infectious-disease information. It is also essential to develop prevention strategies and environmental management plans tailored to the developmental stages and needs of young children. Such targeted approaches can reinforce childcare providers' preventive practices, reduce the spread of infectious diseases in early-childhood education and care environments, and ultimately contribute to better health outcomes for preschool-aged children.

This study has several limitations. First, participants were recruited from an online research panel, which may have introduced selection bias, as panel respondents often differ from the broader population of childcare providers. While the distribution of public facilities and average work experience of the sample aligned well with national trends, the sample also had a higher proportion of master's degrees than the national average (54); thus, generalizability may be limited. Second, although self-efficacy was included, other personal factors influencing infectious-disease prevention behaviors, such as previous experience with infectious diseases and risk perception, were not examined. Third, we used stepwise regression for the exploratory identification of the most influential competency domains. This method risks model overfitting; thus, the findings should be validated using alternative strategies such as hierarchical or LASSO regression in future research. Fourth, although the VIF values indicated no statistical multicollinearity, several competency domains showed high intercorrelations with the outcome variable (*r* > 0.60), suggesting potential conceptual overlap. Therefore, the variance explained by the regression models should be interpreted with caution, as these correlations may partly reflect overlapping constructs. Future research should conduct psychometric testing, including factor analysis, to more clearly distinguish the underlying competency dimensions measured by the practice tool. Lastly, the high levels of environmental management competence observed amid heightened post-COVID-19 awareness may indicate potential ceiling effects, limiting the ability to detect variability and correlations among high performers. Future studies should employ measures with expanded ranges or alternative scoring.

## 5 Conclusion

This study identified the key competencies influencing childcare providers' infection-prevention behaviors in routine and outbreak contexts. Educational guidance and research and empowerment competencies consistently supported stronger preventive practices. Routine prevention was most strongly influenced by educational needs and environmental management, whereas surveillance competency played a central role during outbreaks. These findings clarify which competencies should be prioritized in high-risk childcare environments.

By applying the APIC competency framework and distinguishing routine from outbreak behaviors, we offer a refined understanding of the multidimensional competencies required in childcare settings. Based on our results, facilities should strengthen practical, need-based training—such as handwashing roleplay, routine environmental disinfection, ventilation checklists, and outbreak-response drills—alongside data-driven learning activities.

At the policy level, preparedness may be enhanced by incorporating competency-based assessments into mandatory training and emphasizing educational guidance and surveillance during certification renewal. In summary, this study offers actionable directions for competency-focused training and policy improvements that can reinforce infection prevention and protect the health of young children.

## Data Availability

The datasets used and analyzed during the current study are available from the corresponding author upon reasonable request.
