# Supplementary material for: Competencies influencing childcare providers' infectious-disease prevention in routine and outbreak contexts: a study in South Korea
Source: Front Public Health. 2026 Jan 14;13:1707317. doi: 10.3389/fpubh.2025.1707317 (PMC12847404; doi:10.3389/fpubh.2025.1707317)
Supplement: Supplementary file 1 [file Table_1.docx]

Supplementary Material

**1. Supplementary material 1**

**Supplementary Table 1. Correlation Coefficients of the Study Variables (N = 239)**

| Variables | Practice total | | Everyday prevention practice | | Disease outbreak practice | |
| --- | --- | --- | --- | --- | --- | --- |
|  | r | *p* | r | *p* | r | *p* |
| Self-efficacy in preventing infectious diseases | 0.47^**^ | <0.001 | 0.35^**^ | <0.001 | 0.46^**^ | <0.001 |
| Demand for education on infection prevention and management | 0.55^**^ | <0.001 | 0.62^**^ | <0.001 | 0.47^**^ | <0.001 |
| Competency in preventing infectious diseases | 0.71^**^ | <0.001 | 0.65^**^ | <0.001 | 0.66^**^ | <0.001 |
| Leadership and proficiency | 0.52^**^ | <0.001 | 0.41^**^ | <0.001 | 0.52^**^ | <0.001 |
| Environmental management | 0.53^**^ | <0.001 | 0.62^**^ | <0.001 | 0.46^**^ | <0.001 |
| Knowledge and documentation | 0.59^**^ | <0.001 | 0.56^**^ | <0.001 | 0.55^**^ | <0.001 |
| Surveillance | 0.64^**^ | <0.001 | 0.58^**^ | <0.001 | 0.60^**^ | <0.001 |
| Infection risk management | 0.61^**^ | <0.001 | 0.63^**^ | <0.001 | 0.54^**^ | <0.001 |
| Educational guidance | 0.67^**^ | <0.001 | 0.64^**^ | <0.001 | 0.60^**^ | <0.001 |
| Research and empowerment | 0.63^**^ | <0.001 | 0.53^**^ | <0.001 | 0.60^**^ | <0.001 |
| Personal management | 0.61^**^ | <0.001 | 0.52^**^ | <0.001 | 0.59^**^ | <0.001 |

** <0.001, * <0.05

**2. Supplementary material 2: Survey Instruments and Measurement Tools (Korean version)**

**1) Infectious-disease prevention self-efficacy**

| 질문 내용 | | 아주  자신  있다  (4) | 약간  자신  있다  (3) | 약간  자신  없다  (2) | 아주  자신  없다  (1) |
| --- | --- | --- | --- | --- | --- |
| 1 | 나는 언제 손을 씻어야 하는지 정확히 실천할 수 있다. |  |  |  |  |
| 2 | 나는 손 씻는 방법(6단계)을 정확히 수행할 수 있다. |  |  |  |  |
| 3 | 나는 전문가의 도움이 필요한 감염병 증상을 판별해내 대처할 수 있다. |  |  |  |  |
| 4 | 나는 내가 일하는 어린이집 감염예방 지침에 따라 감염관리를 할 수 있다. |  |  |  |  |
| 5 | 나는 아동을 돌보는 동안, 아동에게 발생할 수 있는 감염병을 예방할 수 있다. |  |  |  |  |
| 6 | 나는 아동이 감염병으로 인해 격리할 상황을 정확하게 판단할 수 있다. |  |  |  |  |
| 7 | 나는 아동에게 어떤 감염병이 의심될 때 부모에게 정확하게 설명할 수 있다. |  |  |  |  |
| 8 | 나는 장난감이나 교구를 소독하는 방법을 정확하게 말할 수 있다. |  |  |  |  |
| 9 | 나는 감염이 우려되는 분비물(기저귀 교환, 설사변, 아동의 침, 콧물을 닦은 휴지)을 처리할 때 다른 아동에게 오염되지 않도록 관리할 수 있다. |  |  |  |  |
| 10 | 나는 어린이집의 쾌적한 환경을 위해 적절한 온도와 습도를 관리할 수 있다. |  |  |  |  |
| 11 | 나는 내가 돌보는 아동의 발달단계에 따라 시행해야 하는  예방접종의 종류를 정확하게 말할 수 있다. |  |  |  |  |
| 12 | 나는 감염예방을 위해 어린이집 방문자를 관리할 수 있다. |  |  |  |  |
| 13 | 나는 아동에게 올바른 손 씻기 방법(6단계)을 지도할 수 있다. |  |  |  |  |
| 14 | 나는 아동에게 올바른 호흡기 질환 예방법을 지도할 수 있다. |  |  |  |  |
| 15 | 나는 아동에게 올바른 화장실 사용법을 지도할 수 있다. |  |  |  |  |

**2) Infectious-disease prevention competency**

| 질문내용 | | 매우 그렇다.  (5) | 그렇다.  (4) | 보통이다.  (3) | 그렇지 않다.  (2) | 전혀 그렇지 않다.  (1) |
| --- | --- | --- | --- | --- | --- | --- |
| 1 | 나는 경험에 의해 아동의 감염병 징후를 신속하게 알아차릴 수 있다. |  |  |  |  |  |
| 2 | 나는 신종 감염병 유행시기의 예측불가한 상황에서도 대처할 자신이 있다. |  |  |  |  |  |
| 3 | 나는 다른 직원들과 아동 감염병 예방 정보 및 해결을 위한 의사소통을 효율적으로 할 수 있다. |  |  |  |  |  |
| 4 | 나는 감염병 예방 업무에 통솔력과 리더십을 갖추고 있다. |  |  |  |  |  |
| 5 | 나는 원내 감염병예방 필수품 (손세척제, 휴지, 마스크, 체온계, 검사키트 등)을 충분히 배치하고 사용할 수 있다. |  |  |  |  |  |
| 6 | 나는 아동이 스스로 손을 씻을 수 있도록 돌보며, 필요시 직접 손을 씻긴다. |  |  |  |  |  |
| 7 | 나는 원내 실내 공기를 수시로(일 3회 이상) 환기한다. |  |  |  |  |  |
| 8 | 나는 원내 실내 적정 온도와 습도를 유지하도록 관리한다. |  |  |  |  |  |
| 9 | 나는 원내 감염관리책임자의 감염병 예방 관리 지시를 따른다. |  |  |  |  |  |
| 10 | 나는 아동기에 주로 발병하는 감염병의 종류에 대해서 알고 있다. |  |  |  |  |  |
| 11 | 나는 아동의 감염병 종류에 따른 징후, 증상에 대해서 알고 있다. |  |  |  |  |  |
| 12 | 나는 수시로 원아의 건강상태를 관찰하고 감염병 관련 자료를 수집, 기록할 수 있다. |  |  |  |  |  |
| 13 | 나는 수집하고 기록된 감염병 관련 자료를 이해하고 그 의미를 알 수 있다. |  |  |  |  |  |
| 14 | 나는 기관의 보육철학과 장단기 보육목표를 고려하여 원내의 감염병 예방 계획을 평가할 수 있다. |  |  |  |  |  |
| 15 | 나는 원내 감염병 예방 관리 프로그램을 위한 자원을 추천할 수 있다. |  |  |  |  |  |
| 16 | 나는 감염병 예방을 위한 원내 인력문제 및 환경개선 방안을 검토하고 제안할 수 있다. |  |  |  |  |  |
| 17 | 나는 원내에 감염병 발생 시, 비상연락체계에 따라 공중보건기관에 연락할 수 있다. |  |  |  |  |  |
| 18 | 나는 원내에 감염병 발생 시, 감염병의 특성(잠복기, 전염가능 시기, 전파 경로 등)을 조사할 수 있다. |  |  |  |  |  |
| 19 | 나의 보육업무 중에 감염병 예방 지침을 준수하는 것은 가장 기본적인 필수 업무이다. |  |  |  |  |  |
| 20 | 나는 감염병 예방 지침을 지키기 어려운 상황에서 다른 사람들에게 도움을 요청할 수 있다. |  |  |  |  |  |
| 21 | 나는 감염전파 위험성이 높은 아동의 신체활동을 인식하고 조정하여 관리할 수 있다. |  |  |  |  |  |
| 22 | 나는 감염전파 위험성이 높은 식사와 간식 시간에 감염병 예방 수칙을 준수하여 관리할 수 있다. |  |  |  |  |  |
| 23 | 나는 개인 아동의 수준(연령, 발달수준 및 건강상태)에 따라 감염병 예방 교육을 할 수 있다. |  |  |  |  |  |
| 24 | 나는 아동이 바람직한 감염병예방 행동(손씻기, 마스크 착용, 이 닦기, 식습관, 기침예절 등)을 형성할 수 있도록 지도할 수 있다. |  |  |  |  |  |
| 25 | 나의 감염병 예방 행동으로 재원아동의 질병 발생을 최소화할 수 있다. |  |  |  |  |  |
| 26 | 나는 감염병 예방 조치(마스크 착용, 거리두기 등)를 고려하여 보육 과정을 개발 및 조정하여 운영할 수 있다. |  |  |  |  |  |
| 27 | 나는 감염병 확진(의심) 원아의 부모에게 상황에 대해 정확하게 설명할 수 있다. |  |  |  |  |  |
| 28 | 나는 아동의 감염병 예방 교육을 위하여 아동에게 그림이나 영상 등을 사용하여 시연할 수 있다. |  |  |  |  |  |
| 29 | 나는 메스컴 등을 통해 보도되는 감염병 관련 연구결과를 자주 숙지한다. |  |  |  |  |  |
| 30 | 나는 나의 감염병 관련 지식이 어느 정도인지 성찰하고 연구결과를 탐색한다. |  |  |  |  |  |
| 31 | 나는 아동기 감염병 관련 조사 및 프로그램 개발 연구에 적극적으로 참여한다. |  |  |  |  |  |
| 32 | 나는 감염병 예방에 대한 지식 및 보육 소양을 갖추기 위한 보완을 지속적으로 하고 있다. |  |  |  |  |  |
| 33 | 나는 알게 된 감염병 관련 연구결과를 보육업무에 효과적으로 적용하기 위해 노력한다. |  |  |  |  |  |
| 34 | 나는 보육교직원, 재원아동 및 재원아동 보호자를 대상으로 감염병 예방 교육을 할 수 있다. |  |  |  |  |  |
| 35 | 나는 모든 보육교직원들(사무원, 관리원, 영양사, 조리사, 차량기사, (보조)교사, 위생원, 외부강사 등)의 감염성 질병에 대한 직업적 노출위험을 인지할 수 있다. |  |  |  |  |  |
| 36 | 나는 다른 보육교직원들(사무원, 관리원, 영양사, 조리사, 차량기사, (보조)교사, 위생원, 외부강사 등)에게 필요한 감염병예방 교육을 권장할 수 있다. |  |  |  |  |  |
| 37 | 나는 다른 보육교직원들(사무원, 관리원, 영양사, 조리사, 차량기사, (보조)교사, 위생원, 외부강사 등)과 감염병 예방을 위하여 정보를 교환하며 협력한다. |  |  |  |  |  |
| 38 | 나는 외부 방문객의 감염병 전파 위험을 통제할 수 있다. |  |  |  |  |  |

**3. Educational needs for infectious-disease prevention**

| 질문 내용 | | 매우 필요하다  (4) | 필요하다  (3) | 필요하지 않다  (2) | 전혀 필요하지 않다  (1) |
| --- | --- | --- | --- | --- | --- |
| 1 | 아동기 주요 감염병 종류 및 증상 |  |  |  |  |
| 2 | 격리해야 하는 감염병과 격리 방법 |  |  |  |  |
| 3 | 감염병 의심 시 관리 방법 |  |  |  |  |
| 4 | 접촉감염 예방 방법 |  |  |  |  |
| 5 | 비말감염 예방 방법 |  |  |  |  |
| 6 | 예방접종 내용 및 접종 시기 |  |  |  |  |
| 7 | 손씻기 방법 및 시기 |  |  |  |  |
| 8 | 유아교육기관에서의 기저귀 관리방법 |  |  |  |  |
| 9 | 분비물 처리 및 폐기 방법 |  |  |  |  |
| 10 | 급식 및 조유관리 방법 |  |  |  |  |
| 11 | 교구/교실 세척 및 소독 방법 |  |  |  |  |
| 12 | 침구소독 및 관리 방법 |  |  |  |  |
| 13 | 방문자 관리방법 |  |  |  |  |
| 14 | 감염병 예방과 관련된 직원관리/ 의사소통 방법 |  |  |  |  |

**4. Infectious-disease prevention practice**

| **질문내용** | | **항상 한다.**  **(4)** | **자주 한다.**  **(3)** | **가끔 한다.**  **(2)** | **전혀 하지 않는다.**  **(1)** |
| --- | --- | --- | --- | --- | --- |
| 1 | 손 위생과 호흡기 예절을 강조한다. |  |  |  |  |
| 2 | 아동이 손을 씻을 때 흐르는 물에 적어도 15초 이상 비누로 씻도록 한다. |  |  |  |  |
| 3 | 장난감, 자주 만지는 지면 및 침구류와 같은 물건들에 대한 청소 및 소독을 강화한다. |  |  |  |  |
| 4 | 아동의 기저귀 교환 후, 또는 용변 보는 것을 도와준 후 손을 씻는다. |  |  |  |  |
| 5 | 식사시간에 아동마다 개별 수저를 사용하도록 한다. |  |  |  |  |
| 6 | 아동의 개별 수저를 주기적으로 소독(열탕, 자외선 살균소독 등) 한다. |  |  |  |  |
| 7 | 설사변이 있는 기저귀, 감염이 우려되는 분비물은 즉시 처리한다. |  |  |  |  |
| 8 | 아동의 등, 하원 과정을 모니터링한다. |  |  |  |  |
| 9 | 아동의 결석을 모니터링한다. |  |  |  |  |
| 10 | 감염병 관리와 관련된 물품상태를 모니터링하고 보충 가능성을 확보한다. |  |  |  |  |
| 11 | 공기 청정기 설치 등 환기를 증대하기 위해 노력한다. |  |  |  |  |
| 12 | 환기가 잘되지 않는 공간을 감소시키기 위해 노력한다. |  |  |  |  |
| 13 | 교실 별 그룹화를 통한 그룹간 감염원 노출을 최소화한다. |  |  |  |  |
| 14 | 원내 감염병 관리 담당자를 지정(요청)한다. |  |  |  |  |
| 15 | 감염병 발생 시 대응 프로토콜에 대해 교육받았다. |  |  |  |  |
| 16 | 감염병 발생 시 감염이 의심되는 아동 또는 교직원이 집에 머무르도록 한다. |  |  |  |  |
| 17 | 감염 의심 아동 또는 교직원의 증상을 감시한다. |  |  |  |  |
| 18 | 유아교육시설에 들어오기 전 모든 교직원 및 아동의 증상 및 감염징후에 대한 매일의 검사를 실시한다. |  |  |  |  |
| 19 | 아동 또는 교직원이 감염 증상이 보일 시 표준 운영 절차에 따라 행동한다. |  |  |  |  |
| 20 | 격리실의 위치와 사용시점에 대해 알고 있다. |  |  |  |  |
| 21 | 감염병 발생 사례에 대한 보건 당국 보고를 계획한다. |  |  |  |  |
| 22 | 감염병 별 의료연계, 검사에 대한 지침을 배포한다. |  |  |  |  |
| 23 | 감염병 별 격리 지침이나 안내서를 배포한다. |  |  |  |  |
| 24 | 감염병의 잠복기간에 따른 격리 기간을 요구한다. |  |  |  |  |
| 25 | 유아교육시설 교직원을 위한 유연한 감염병 휴가 정책을 적용(요청)한다. |  |  |  |  |
| 26 | 아동의 보호자, 유아교사 및 기타 직원을 대상으로 표준 운영 절차 및 완화 조치에 대한 교육을 지속적으로 실시한다. |  |  |  |  |
| 27 | 감염병 의심 증상 파악을 위한 감시 및 교육을 실시한다. |  |  |  |  |
| 28 | 유아교사 및 기타 유아교육시설의 직원에게 감염병 관리와 관련된 적절한 개인 보호 장비(예: 마스크, 얼굴 보호대 및 가운) 제공한다. |  |  |  |  |
| 29 | 아동의 보호자, 유아교직원이 감염 예방 전략을 준수하도록 장려한다. |  |  |  |  |
| 30 | 감염병 고위험 군에 해당하거나, 아이가 있는 유아교육시설의 교직원을 위한 유연한 휴가정책을 수립한다. |  |  |  |  |
| 31 | 유연한 근무 시간 및 당번 근무제를 시행한다. |  |  |  |  |
| 32 | 감염병 위험이 높은 교직원을 위한 원격 근무에 대한 선택권을 제공(요청)한다. |  |  |  |  |
